# Medical Health Records-Based Mild Cognitive Impairment (MCI) Prediction for Effective Dementia Care

**DOI:** 10.3390/ijerph18179223

**Published:** 2021-09-01

**Authors:** Soo-Jin Lim, Zoonky Lee, Lee-Nam Kwon, Hong-Woo Chun

**Affiliations:** 1 Convergence Research Center for Diagnosis, Treatment and Care System of Dementia, Korea Institute of Science and Technology, Seoul 02792, Korea; sjlim@kist.re.kr (S.-J.L.); ynkwon@kist.re.kr (L.-N.K.); 2 Future Technology Analysis Center, Korea Institute of Science and Technology Information, Seoul 02456, Korea; 3 Graduate School of Information, Yonsei University, Seoul 03722, Korea; zlee@yonsei.ac.kr

**Keywords:** dementia early prediction, machine learning, medical records, mild cognitive impairment prediction, senior cohort

## Abstract

Dementia is a cognitive impairment that poses a global threat. Current dementia treatments slow the progression of the disease. The timing of starting such treatment markedly affects the effectiveness of the treatment. Some experts mentioned that the optimal timing for starting the currently available treatment in order to delay progression to dementia is the mild cognitive impairment stage, which is the prior stage of dementia. However, medical records are typically only available at a later stage, i.e., from the early or middle stage of dementia. In order to address this limitation, this study developed a model using national health information data from 5 years prior, to predict dementia development 5 years in the future. The Senior Cohort Database, comprising 550,000 samples, were used for model development. The F-measure of the model predicting dementia development after a 5-year incubation period was 77.38%. Models for a 1- and 3-year incubation period were also developed for comparative analysis of dementia risk factors. The three models had some risk factors in common, but also had unique risk factors, depending on the stage. For the common risk factors, a difference in disease severity was confirmed. These findings indicate that the diagnostic criteria and treatment strategy for dementia should differ depending on the timing. Furthermore, since the results of this study present new dementia risk factors that have not been reported previously, this study may also contribute to identification of new dementia risk factors.

## 1. Introduction

With the aging of the population, early diagnosis and timely treatment of dementia are among the key focus areas in medicine. Early treatment of dementia can slow the disease progression, whereas a delay in treatment leads to reduced efficacy of medication and shortens the period during which the patient can benefit from the effect of treatment [1]. It has been concluded that the earliest point of diagnosis at which treatment is effective is mild cognitive impairment (MCI), which is considered to represent the early stage of dementia [2]. MCI refers to a state in which the patient experiences a decline in short-term memory, with forgetfulness regarding recent events, but with no significant impairment in everyday functioning [3]. Given that taking early preventive measures at the stage of MCI delays the progression to dementia, researchers have increasingly emphasized the importance of early prediction of MCI [4].

Several studies have developed techniques for the early diagnosis of dementia based on brain magnetic resonance imaging (MRI), which has presented excellent performance, with area under the ROC curve (AUC) values of 98% for Alzheimer’s disease and 87% for MCI in a previous study [5]. Positron emission tomography (PET), the most commonly employed neuroimaging tool for dementia diagnosis, can demonstrate neurometabolic changes and the presence of amyloid β, a dementia-related protein in the brain. However, this modality is expensive to perform, and requires ionizing radiation, as well as installation of special equipment, thus limiting its clinical utilization [4,5,6]. Brain magnetic resonance imaging (MRI) scans are cost-effective relative to PET scans and have the advantage of not involving radiation exposure. However, its diagnostic performance has been limited to late-stage dementia and is less effective in the early detection of the disease.

The collection of neuroimaging data described above has practical difficulties [7]. However, in Korea, the National Health Insurance Service (NHIS) which manages healthcare information for the whole Korean population, stores and manages such data, consequently, no additional time and effort is required for data collection. In addition, the NHIS data are not obtained using a cross-sectional survey of short-term analysis for a small group, but consists of cohort-type data. Most previous studies using NHIS data have focused on the association between a single disease and dementia, and few studies have investigated the causes of dementia by considering multiple factors [7,8,9].

Moreover, examinations and tests in patients who show that abnormal behavior along with memory impairment are generally not performed in the early stage of MCI, but rather in the later stage of MCI or when dementia has already developed [3,4]. Consequently, most existing dementia prediction studies based on NHIS data in fact report predictions of mild dementia, rather than early prediction in the true sense. Neurodegenerative disorders, which cause dementia, have been reported to have an onset approximately 20 years before dementia onset, while the onset of MCI occurs about 5 years prior to dementia onset [2,3,4]. If the characteristics of MCI patients can be identified early and if this information can be used for prediction of risk of further progression to dementia in the distant future, it would be of significant use in early diagnosis.

Therefore, in this study, we aimed to develop a model for predicting the risk of dementia development, 5 years in the future, during the true early stage of MCI, using machine learning. We used the NHIS health information data, assessed a wide range of factors when training the model, and retained only predictors with a significant effect in the final prediction model.

## 2. Materials and Methods

### 2.1. Data Source

The NHIS is a quasi-governmental organization responsible for the public health of the whole Korean population, and it manages and operates the National Health Insurance (NHI), which protects the general public against the risk of disease, and the Long-Term Care Insurance, which aims to ensure a comfortable life in old age [10]. According to the NHIS, the number of Koreans covered by the NHI in 2019 was 51,391,447. Given the registered number of residents in Korea, this number indicates that almost all Koreans are covered by the NHI, and that the NHIS database is representative of the total population of Korea [11]. In addition, the NHIS operates the National Health Insurance Sharing Service (NHISS), which supports policy and academic research using the national health information data. The Sample Research Database (DB) held by the NHISS includes standardized and de-identified datasets for use by academic research by sampling high-demand data from the pool of big data of the NHIS [12,13]. There are five types of DBs: The Sample Cohort DB, Medical Check-up Cohort DB, Senior Cohort DB, Infant Medical DB, and Working Women DB. Among these DBs, the Senior Cohort DB was used in this study for prediction of dementia.

The Senior Cohort DB is a research dataset constructed to support research targeting older individuals [10,11]. In 2002, 10% of the 5.5 million subjects aged 60 years or older, who were eligible for NHI coverage or medical aid, were sampled using simple random sampling, resulting in construction of a database of about 550,000 older people from the Korean population [14]. The data in this database are in cohort format and represent a 14-year data collection period (2002–2015). In this study, data from a 12-year period (2002–2013) within this 14-year period were used. The Senior Cohort DB consists of the Participant Insurance Eligibility DB (PIE-DB), Medical Treatment DB (MT-DB), General Health Examination DB (GHE-DB), Medical Care Institution DB (MCI-DB), and Long-term Care Insurance DB (LCI-DB). The PIE-DB contains sociodemographic information, such as sex, age, and income level, and the MT-DB contains information on diseases for which the senior subjects have been treated through hospital visits. The GHE-DB contains the main outcomes of health examination and lifestyle data collected by an interview. The MCI-DB contains information on the current status of long-term care institutions, and the LCI-DB contains information related to the application and use of long-term care services by older people. All DBs can serve as instrumental information for predicting dementia. However, in this study, only the PIE-DB and MT-DB were used, since the main focus of the research was on identifying risk factors causing dementia.

### 2.2. Study Population

To develop a model for predicting dementia 5 years in the future (the 5-year incubation period model), which was the key objective of this study, models for predicting dementia after 1 and 3 years (the 1- and 3-year incubation period models, respectively) were also developed for comparison against the 5-year incubation period model. The incubation period used in this study refers to the time during which the patient is in the MCI stage, before being diagnosed with dementia. The 2007–2012 data were used for training of each prediction model, and dementia status was determined using the data from 2013. In other words, in constructing the dataset of all incubation period models, 2-year hospital records were used. The 5-year incubation period model used hospital records from 2007–2008, the 3-year incubation period model used hospital records from 2009–2010, and the 1-year incubation period model used hospital records from 2011–2012 for model training.

**Figure 1:**
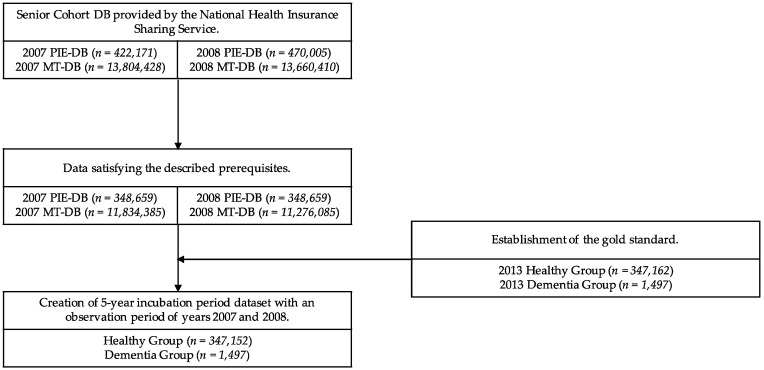
Flowchart of the study population. Abbreviations: PIE-DB: Participant Insurance Eligibility Database; MT-DB: Medical Treatment Database.

Next, there were two inclusion criteria for constructing the dataset. First, only patients with records of treatment at least once in each year from 2007 to 2013 were included in the dataset. This criterion was established to track how the medical records changed before 2013, when the patient was diagnosed with dementia. The second criterion was that patients who had never been diagnosed with dementia from 2007 to 2012 were included in the dataset. Since the model was aimed at prediction of MCI, use of data from healthy subjects in the same period of observation was required. Samples of data satisfying both of these criteria were used to construct the dataset. The process of class assignment for the healthy group and the dementia group in constructing the gold standard is described in detail in Section 2.3. When the data class assignment was completed, the gold standard to be used for modeling was finally determined. Figure 1 describes the process of dataset construction and the number of data points per case for the 5-year incubation period model.

### 2.3. Gold Standard

The gold standard datasets are the datasets used for training the model. First, feature sets were constructed for each model using data from 2007 to 2012. Next, the healthy group and the dementia group were classified based on the dementia diagnosis status in the principal and secondary diagnostic features in the MT-DB, according to 2013 data. The principal diagnosis and secondary diagnosis columns were based on the code system of Korean Standard Classification of Diseases (KCD), which was established in consideration of Korean circumstances, based on the International Classification of Diseases published by the World Health Organization [15]. The revision of the disease classification of the KCD is ongoing, and since this study used data from 2002 to 2013 for the analysis, the 6th edition of the KCD was used [16]. The disease classification consists of a systematic classification system (major group, sub-major group, minor group, detailed group, and sub-detailed group), and diseases are classified into sub-detailed groups in the Senior Cohort DB. For classification into dementia groups, five types of detailed group classification codes, which are “Dementia in Alzheimer’s disease (F00)”, “Vascular dementia (F01)”, “Dementia in other diseases classified elsewhere (F02)”, “Unspecified dementia (F03)”, and “Alzheimer’s disease (G30)”, were used. These codes, which have been used in several previous studies on dementia, were used in the classification in this study (Table 1) [10,11,17].

### 2.4. Sociodemographic Features

Sociodemographic features included sex, age, region of residence, health insurance type, household income level, and disability registration information. Additionally, the educational level was used and consisted of seven features. Participants were classified into six age groups, defined at intervals of 5 years from the age of 65 years. The region of residence was divided into 16 districts according to administrative districts, which have been reorganized into urban and rural. The health insurance type was classified into three categories. Household income level was classified into 11 levels, which have been reorganized into three groups. As for disability registration information, levels 1–2 of severe disability and levels 3–6 of minor disability were assigned to separate categories (Table 2). A number of studies have presented educational level as a dementia risk factor, and have found that a lower educational level was associated with a higher risk of dementia [18,19]. In this study, an educational level feature, which is not included in the Senior Cohort DB, was created, in addition to the existing features of the health insurance type and household income level.

### 2.5. Disease Severity Classification Algorithm

**Figure 2:**
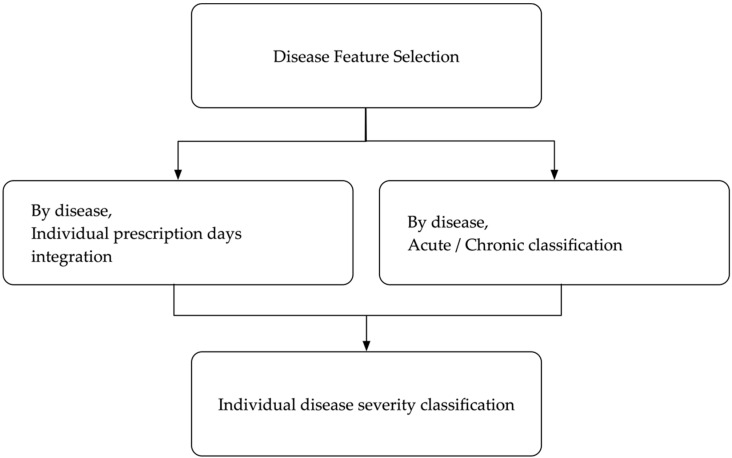
Flowchart of the disease severity classification algorithm.

In this study, an algorithm for classifying disease severity was developed. Most of the existing techniques in previous studies considered the cumulative incidence as the number of disease occurrences over a certain period among those who were likely to develop the disease during the observation period, using only hospital records, without a severity analysis. In the present study, the disease was analyzed based on the date of prescription and the features were constructed in consideration of disease severity. Figure 2 describes the flowchart of the disease severity classification algorithm.

First, since the classification system of diseases based on sub-detailed group classification involves excessively detailed classification for analysis, it was processed into sub-major group classification. Consequently, 215 disease features were generated using the disease diagnosis code. Second, the severity of each disease was calculated using the “date of prescription” and the “total number of prescription days for 2 years” features, without duplication in the process. Third, since this approach does not reflect differences in the characteristics of the disease, the study reflected the disease characteristics according to the time of onset, and divided the disease into acute and chronic conditions. If the classification was based on chronic diseases with a relatively long treatment period, the effect of the diseases with a short treatment period was reduced [20]. To develop rules through which the diseases could be classified into acute and chronic conditions, criteria were established based on the distribution map. If the number of cases with a total number of prescription days of 11 days or longer was ≤3000, the disease was classified as an acute disease, and if it was >3000, the disease was classified as a chronic disease. Although there was no clear consensus in the medical research community in terms of classification between acute and chronic diseases, the criteria created in this study were compared with those of some published major chronic diseases for validation [21,22,23,24]. This comparison confirmed that all published chronic diseases were included in the chronic disease classification used in this study. Finally, to analyze the change in disease severity between the 5-year incubation period model and the 1- and 3-year incubation period models, each disease’s severity was divided into four stages: “Preclinical,” “minor,” “severe,” and “critical.” Based on the period of 2 years, “preclinical” (0 days) for acute disease indicated a case without any treatment, and “minor” (1–7 days) indicated a case of a once-off treatment or treatment within 1 week. “Severe” (8–30 days) indicated treatment for 1 month, and “critical” (≥31 days) indicated treatment for more than 1 month. For chronic diseases, “preclinical” (0 days) indicated a case of no treatment experience, “minor” (1–30 days) indicated that the patient had been prescribed medication for 1 month, “severe” (31–210 days) indicated that the patient had been prescribed medication for half a year, while “critical” (≥211 days) indicated a case of treatment for ≥1 year (Table 3). Accordingly, by converting the total number of prescription days (data in numbers) into categorical data, we created the criteria for disease severity classification.

### 2.6. Feature Selection

In the process of machine learning, not all features represent important information. It is necessary to remove redundant and irrelevant variables from the data and select only the input variables that are closely related to the target variable [25]. There are various methods of feature selection, depending on the methods of machine learning. Supervised learning mainly uses the filter method and the wrapper method [26]. The filter method measures the relevance of features by correlation with the dependent variables. In contrast, the wrapper method creates a set of optimized features by performing continuous tests on feature subsets. In this study, there were seven sociodemographic variables and 215 disease variables. We used the wrapper method, since identification of the best feature combination is required in datasets with numerous features. The wrapper method includes forward selection, backward elimination, and stepwise selection. Forward selection starts with no variables and adds the most important variable at every iteration step of the modeling. This process is repeated until there is no further improvement in model performance. Backward elimination starts with all variables and removes the least significant variable, one by one, to improve the performance of the model. Stepwise selection is a combination of forward selection and backward elimination. Using all three types of feature selection methods, the significance level was set to 0.05 and only variables whose *p*-value was less than or equal to the set significance level were selected. In this study, the input variables were constructed as the combination of features obtained using all three methods rather than only one method. Consequently, the number of features constituting each incubation period model decreased with the increase in time from the year of dementia diagnosis (Table 4).

## 3. Results and Discussion

### 3.1. Modeling

In this section, the characteristics of the three algorithms that showed the best performance among various classification models, i.e., random forest, support vector machine (SVM), and multi-layer perceptron (MLP), are outlined. First, with the random forest algorithm, random sampling of data is performed in a given dataset to make multiple decision trees. Based on the prediction result of each decision tree, the final prediction is determined by a majority vote [27,28]. Therefore, the random forest is a combination of several decision trees and can avoid overfitting. Moreover, the algorithm shows high stability [28]. In this study, 200 decision trees were generated in the 5-year incubation period model for analysis. Second, the SVM classifies data that are difficult to distinguish in two dimensions using hyperplanes in a finite dimensional space [29]. Accurate classification and prediction of data are achieved through a hyperplane with a margin that maximizes the separation between data from a given dataset that needs to be classified [30]. Even when linear classification of the data is not possible with the SVM method, nonlinear classification of data can be performed using a kernel function. In this study, SVM classification was performed using the radial basis function kernel, a nonlinear kernel function, in the 5-year incubation period model. Third, the MLP algorithm uses the back propagation algorithm that reflects the difference between the target value and the output value in the hidden layer and adjusts the weight for training a neural network [31]. The accuracy improves with iteration of this process, and thus the number of epochs has a significant impact on the performance. In this study, two hidden layers were created in the 5-year incubation period model and 26 and 22 nodes were assigned to each layer. Additionally, a hyperbolic tangent function was used as the activation function in both hidden layers.

The random forest is the simplest to use, and shows excellent performance and can appropriately identify a robust model. The SVM is also an excellent approach that has been sufficiently verified and presents excellent classification performance, regardless of the size or complexity of the data. On the other hand, the MLP does not require as much attention to feature engineering as other approaches, but it is more suitable for processing relatively large amounts of data with complex processing, such as image classification and speech recognition.

Prior to modeling with a machine learning algorithm, the data distribution between the healthy group and dementia group was imbalanced, which needed to be addressed first [32]. When prediction is performed with imbalanced sets of data, overfitting may occur. This is due to the fact that the model attempts to predict classes with higher weights. Thus, precision may be increased, but the recall of classes with small distributions is decreased. Methods for resolving data imbalance include oversampling and undersampling. In this study, random undersampling was performed with the healthy group data with high distribution to balance the number of cases between the healthy group and the dementia group.

Data that have undergone all processing can be divided into training and test data, but if this process is performed alone, overfitting may also occur [33]. This is due to the fact that, when the evaluation is performed with fixed training and test datasets, the model is compelled to produce a biased outcome in which the optimal performance is achieved only for the test data. In this study, to address this problem, stratified K-fold cross-validation was used to create 10-fold cross-validation, and the performance of the algorithm was evaluated. In addition, to improve model performance, hyper-parameter optimization was performed using a grid search.

### 3.2. Performance Measures

The proposed approach evaluates the models by five measures as follows: Precision, recall, F-measure, accuracy, and AUC. If a healthy case is classified as a dementia case, it can be re-assigned as a healthy case by performing additional tests, but when a dementia case is incorrectly classified as a healthy case, the appropriate treatment timing may be missed. In this regard, recall, precision, and accuracy are important indicators in the problem of disease prediction [34]. The F-measure, an index that considers both precision and recall, is obtained by calculating the harmonic mean of precision and recall. In our study, the F-measure was determined by adjusting the threshold so that the recall and precision values did not exhibit bias to one side. The AUC refers to the area under the ROC curve, which is a graph showing the performance of a classification model at all thresholds [35]. The equations of the evaluation measures are as follows:

(1) $$\mathrm{Precision}=\frac{\mathrm{DD}}{\mathrm{HD}+\mathrm{DD}}$$

(2) $$\mathrm{Recall}=\frac{\mathrm{DD}}{\mathrm{DH}+\mathrm{DD}}$$

(3) $$\mathrm{Accuracy}=\frac{\mathrm{HH}+\mathrm{DD}}{\mathrm{HD}+\mathrm{HH}+\mathrm{DD}+\mathrm{DH}}$$

(4) $$F-\mathrm{measure}=\frac{2\;\times \;\mathrm{Precision}\;\times \;\mathrm{Recall}}{\left(\mathrm{Precision}+\mathrm{Recall}\right)}$$

where HH: Healthy classified as healthy, HD: Healthy classified as dementia, DD: Dementia classified as dementia, DH: Dementia classified as healthy.

### 3.3. Evaluation Results

Among the performance indicators used in the classification model, this study used the F-measure as the main performance indicator of the model under evaluation. As a result of the experiments outlined in Table 5, the best F-measure performance was obtained when the random forest algorithm was used in all incubation period models.

The random forest method is optimized for imbalanced data, as compared to the SVM, and thus it performed a more accurate prediction with the algorithm, which was more favorable to the overfitting problem. Furthermore, since forward propagation is sufficient for NHIS health information data without the need for back propagation, the performance with MLP modeling was slightly lower than that of other algorithms.

Each incubation period model showed good prediction performance, in the order of the 1-, 3-, and 5-year incubation period. The 5-year incubation period model, predicting a more distant future, yielded an F-measure of 77.38%, and the 1-year incubation period model, predicting the near future, produced an F-measure of 90.71%. Although the results of the distant future prediction model naturally showed lower performance than the near future model, it yielded encouraging results. This indicated that dementia can be predicted with high accuracy, even when only NHIS health information data from 5 years earlier are used for prediction of the stage of MCI.

Furthermore, to show the validity of the acute and chronic classification method applied to the disease severity classification algorithm, the model’s performance was compared before and after disease classification using data from the 5-year incubation period model. Table 6 shows that the F-measure value was improved by 2.26% when the classification algorithm was applied. Thus, higher predictive performance can be achieved when a model is developed considering the characteristics of the disease, rather than only the number of days of prescription.

### 3.4. Important Features

To investigate the importance of features for each incubation period model, permutation feature importance, a commonly used method, was adopted in this study. Permutation feature importance is a method of determining the importance of a feature according to how much it affects performance loss when the feature is not included in the model [36]. The feature importance was examined when the random forest algorithm, the algorithm with the highest performance among the three machine learning methods, was used. Table 7 presents dementia risk factors and their ranking for each incubation period model. The number in parentheses in front of each dementia risk factor in the table indicates the ranking of the dementia risk factor: The higher the ranking (the closer the ranking value is to 1), the more important is the feature. As the incubation period increased from 1 to 5 years, the number of extracted risk factors decreased. This indicated that, as the year of dementia diagnosis approached, the number of abnormal symptoms increased, resulting in more risk factors. However, in the prediction of a more distant future, there are fewer abnormal symptoms that can be identified, and consequently fewer risk factors are identified.

Nevertheless, there were features that were common among the models, as well as those that differed among the models. Table 8 shows only the non-overlapping factors of the 1- and 5-year models. Considering these factors, the appropriate treatment will need to be provided timeously for each period.

Risk factors that have not been identified in previous studies and are newly presented in this study are outlined in Table 9. To determine the publication status in the previous studies, the Named Entity Recognition (NER) method, developed in the biomedical domain, was used [37]. The NER method is a tool that recognizes disease, gene/protein, DNA/RNA, drug, compound, and symptoms, and was shown to have an F-measure of 71.37% in a previous study. We performed NER on all relevant papers identified in PubMed. If the terms “Dementia” or “Alzheimer” and the risk factor candidates proposed in this study appeared together in one paragraph, it was determined as a previously identified risk factor. In this manner, although the risk factors that have been identified as newly proposed predictors of dementia require further verification, the method proposed in this study simplifies and shortens the process of discovering new dementia risk factors.

### 3.5. Changes in the Distribution of Common Risk Factors

Even in the case of the common risk factors of the 1- and 5-year incubation period models, it is judged that there would be differences in disease severity, depending on the dementia incubation period, and therefore, in order to analyze the disease severity of each patient, the severity was calculated for all diseases and changes in the distribution of the number of patients were analyzed. Common risk factors are shown in Table 10, and all factors were disease-related factors, except for “Age”, “Sex”, and “Educational level”.

Changes in the severity distribution of chronic disease factors are presented in Table 11. For “Organic, including symptomatic mental disorders”, “Cerebral palsy and other paralytic syndromes”, and “Mood (affective) disorders”, the number of patients with “minor”, “severe”, and “critical” conditions was increased in the 1-year incubation period model compared to the 5-year incubation period model. For “Metabolic disorders”, only the number of “minor” patients showed a slight increase, but there were no other notable changes. On the other hand, in the case of “Disorders of the lens” and “Disorders of the conjunctiva”, the number of “preclinical” patients actually increased during this period. That is, “Organic, including symptomatic mental disorders” showed increasing severity as the onset of dementia approached, whereas “Disorders of the lens” and “Disorders of the conjunctiva” showed symptom alleviation. Among the six chronic diseases, “Organic, including symptomatic mental disorders”, which showed the most rapid change in symptoms, refers to a type of mental and behavioral disorder and corresponds to a sub-major group category of the KCD that includes codes F00–F09. Except for the detailed group classification code that was used to classify the dementia group in the gold standard creation stage, codes F04–F07 and F09 correspond to “Organic, including symptomatic mental disorders.” The diseases corresponding to codes F04–07 and F09 include “Organic amnestic syndrome, not induced by alcohol and other psychoactive substances”, “Delirium, not induced by alcohol and other psychoactive substances”, “Other mental disorders due to brain damage and dysfunction and to physical disease”, “Personality and behavioral disorders due to brain disease, damage, and dysfunction”, and “Unspecified organic or symptomatic mental disorder”. These five disease factors, together, are determinants that can increase the prevalence of dementia and should be carefully managed in old age to prevent and delay dementia onset.

Next, changes in the severity distribution of acute disease factors are outlined in Table 12. “Mental and behavioral disorders due to psychoactive substance use” and “Pediculosis, acariasis, and other infestations” showed an increase in the number of “minor” and “severe” patients. On the other hand, “Acute upper respiratory infections” showed an increase in the number of “preclinical” patients. The distribution of all factors in acute diseases showed little change as compared to that in chronic diseases.

Thus, even in the case of these common dementia risk factors, our results confirmed that the severity levels of chronic and acute diseases differ, depending on the dementia incubation period. Consequently, our dementia prediction models were developed in consideration of these changes.

## 4. Conclusions

In this study, a dementia prediction model was developed using health information data from the NHIS, in which most of the Korean population have been registered. For prediction of dementia development in the distant future, data from 5 years previously in the true early stage of MCI, which is the optimal time for effective dementia treatment, were used for model development. This is due to the fact that the NHIS health information data usually record dementia data from the late stage of MCI or early stage of dementia, which is about 5 years after the early stage of MCI. The main results and applications of the study are presented as follows.

First, a model for predicting dementia in the distant future (5 years later) was developed using a machine learning algorithm, which showed promising performance (F-measure 77.38%) as compared with the performance of the near-future model (1 year later; F-measure 90.71%). Since the model for predicting dementia with data from 1 year earlier usually represents the middle or late stage of MCI or the early stage of dementia, more dementia-related symptoms are present during the early stage of MCI. Consequently, the performance of a near-future prediction model is naturally high. On the other hand, since the distant-future model predicted dementia using NHIS health information data from 5 years earlier, a time when symptoms are rare, the performance of the model, although lower than that of the near-future model, has significant implications.

Second, risk factors affecting dementia prediction were presented in terms of different incubation periods. Even with common risk factors, it was judged that there would be differences in the severity of the disease according to the incubation period model, and thus changes in the distribution of disease severity were analyzed and compared between models. In this way, information on dementia risk factors and their severity, which are different for each incubation period model, can be used as the optimal criteria for dementia treatment according to the relevant period.

Third, new dementia risk factors, indicating a high risk of developing dementia, were presented, which are different from those that have already been reported to be predictors of dementia in previous studies. If the clinical applicability of the newly proposed risk factors are verified, it will be possible to use them as therapeutic targets for early diagnosis of dementia or development of control methods. The method used here for identification of novel risk factors are expected to facilitate the discovery process of additional new dementia risk factors.

Despite these contributions, the current proposed approach has a limitation. It deals with only the old medical records, so it cannot consider the new environments such as COVID-19. The classification performance would be improved if the new conditions can be considered. Therefore, we will make an agreement with several hospitals about the use of new information for patients and will deal with not only old medical history but also new environments in the next research.

This study is representative of the Korean population and identified risk factors for dementia, according to the incubation period. The findings of this study can be evaluated as data of instrumental value for treatment of dementia disease. In the future, dementia risk factors identified for each incubation period that are verified can be used as baseline data for actual clinical trials. In addition, development of an early dementia prediction model is planned by additional utilization of a range of NHIS health information data, such as the GHE-DB, which contains information on the main health examinations that patients have undergone, and the MCI-DB, which contains information on long-term care institutions, among the data provided by NHISS.

## Figures and Tables

**Table 1:** Dementia classification code.

Code	Diagnosis
F00	Dementia in Alzheimer’s disease
F00.0	Dementia in Alzheimer’s disease with early onset
F00.1	Dementia in Alzheimer’s disease with late onset
F00.2	Dementia in Alzheimer’s disease, atypical, or mixed type
F00.9	Dementia in Alzheimer’s disease, unspecified
F01	Vascular dementia
F01.0	Vascular dementia of acute onset
F01.1	Multi-infarct dementia
F01.2	Subcortical vascular dementia
F01.3	Mixed cortical and subcortical vascular dementia
F01.8	Other vascular dementia
F01.9	Vascular dementia, unspecified
F02	Dementia in other diseases classified elsewhere
F02.0	Dementia in Pick’s disease
F02.1	Dementia in Creutzfeldt Jakob disease
F02.2	Dementia in Huntington’s disease
F02.3	Dementia in Parkinson’s disease
F02.4	Dementia in human immunodeficiency virus (HIV) disease
F02.8	Dementia in other specified diseases classified elsewhere
F03	Unspecified dementia
G30	Alzheimer’s disease
G30.0	Alzheimer’s disease with early onset
G30.1	Alzheimer’s disease with late onset
G30.8	Other Alzheimer’s disease
G30.9	Alzheimer’s disease, unspecified

**Table 2:** Feature engineering on categorical data.

Feature	Value
Region of residence	(1) Seoul, Busan, Daegu, Incheon, Gwangju, Daejeon, and Ulsan, (2) Gyeonggi, Gangwon, Chungcheongbuk, Chungcheongnam, Jeollabuk, Jeollanam, Gyeongsangbuk, Gyeongsangnam, and Jeju
Health insurance type	(1) Medical aid beneficiaries, (2) Locally-provided NHI policy holders, (3) Employment-based NHI policy holders
Household income level	(1) 0 to 3rd deciles, (2) 4th to 7th deciles, (3) 8th to 10th deciles
Disability registration information	(1) Normal, (2) Levels 1–2 of severe disability and levels 3–6 of minor disability

**Table 3:** Classification of disease’s severity.

Stage	Acute Disease (Day)	Chronic Disease (Day)
Preclinical	0	0
Minor	1–7	1–30
Severe	8–30	31–210
Critical	≥31	≥211

**Table 4:** The number of features composed for each incubation period model.

Method	IP 1-Year	IP 3-Year	IP 5-Year
Forward selection	32	29	22
Backward elimination	38	32	23
Stepwise selection	36	29	23
Features finally used	39	32	25

Abbreviations: IP: Incubation Period.

**Table 5:** Performance with application of the machine learning algorithms to each incubation period model.

Incubation Period	Classifier	Precision (%)	Recall (%)	Accuracy (%)	F-Measure (%)	AUC (%)
1-year	RF	87.08	94.65	90.32	**90.71**	95.17
	SVM	89.78	90.05	89.90	89.86	95.68
	MLP	87.86	91.97	89.65	89.87	94.21
3-year	RF	73.55	89.30	78.63	**80.66**	85.37
	SVM	79.34	78.64	79.04	78.93	86.58
	MLP	74.07	86.96	78.30	80.00	84.81
5-year	RF	75.88	78.93	76.96	**77.38**	81.76
	SVM	73.67	76.13	74.49	74.79	82.19
	MLP	71.39	82.61	74.79	76.59	81.77

Note: The F-measure values shown in bold indicate the method with the best performance. Abbreviations: RF: Random Forest; SVM: Support Vector Machine; MLP: Multi-Layer Perceptron; AUC: Area Under the ROC Curve.

**Table 6:** Comparison of the performance of the 5-year incubation period model before and after the disease classification.

Classifier	Disease Classification	Precision (%)	Recall (%)	Accuracy (%)	F-Measure (%)	AUC (%)
Random forest	Before	72.22	78.26	74.12	75.12	80.81
	After	75.88	78.93	76.96	77.38	81.76

**Table 7:** Dementia risk factors according to the incubation period of models.

Incubation Period	Dementia Risk Factors and Ranking
1-year	(1) Organic, including symptomatic mental disorders, (2) Age, (3) Hypertensive diseases, (4) Diseases of oesophagus, stomach, and duodenum, (5) Acute upper respiratory infections, (6) Soft tissue disorders, (7) Other acute lower respiratory infections, (8) Metabolic disorders, (9) Sex, (10) Disorders of lens, (11) Cerebrovascular diseases, (12) Disorders of conjunctiva, (13) Dermatitis and eczema, (14) Educational level, (15) Mood (affective) disorders, (16) Health insurance type, (17) Mycoses, (18) Other diseases of the urinary system, (19) Osteopathies and chondropathies, (20) Diseases of liver, (21) Diseases of inner ear, (22) Cerebral palsy and other paralytic syndromes, (23) Abnormal findings on diagnostic imaging and in functional studies, without diagnosis, (24) Polyneuropathies and other disorders of the peripheral nervous system, (25) Injuries to the head, (26) Injuries to the hip and thigh, (27) Noninfective enteritis and colitis, (28) Aplastic and other anemias, (29) Disease Name of Oriental Medicine, (30) Mental and behavioral disorders due to psychoactive substance use, (31) Other bacterial diseases, (32) Pediculosis, acariasis, and other infestations, (33) Sequelae of infectious and parasitic diseases, (34) Abnormal findings on examination of urine, without diagnosis, (35) Demyelinating diseases of the central nervous system, (36) Poisoning by drugs, medicaments, and biological substances, (37) Inflammatory diseases of the central nervous system, (38) Disease of appendix, (39) Other congenital malformations of the digestive system.
3-year	(1) Age, (2) Organic, including symptomatic mental disorders, (3) Sex, (4) Acute upper respiratory infections, (5) Other acute lower respiratory infections, (6) Diseases of oesophagus, stomach, and duodenum, (7) Metabolic disorders, (8) Disorders of eyelid, lacrimal system, and orbit, (9) Disorders of conjunctiva, (10) Household income level, (11) Osteopathies and chondropathies, (12) Mycoses, (13) Mood (affective) disorders, (14) Injuries to the thorax, (15) Injuries to the head, (16) Other degenerative diseases of the nervous system, (17) Persons with potential health hazards related to family and personal history and certain conditions influencing health status, (18) Human immunodeficiency virus (HIV) disease, (19) Disorders of thyroid gland, (20) Noninfective enteritis and colitis, (21) Cerebral palsy and other paralytic syndromes, (22) Abnormal findings on examination of urine, without diagnosis, (23) Disability registration information, (24) Disorders of skin appendages, (25) Urolithiasis, (26) Symptoms and signs involving the nervous and musculoskeletal systems, (27) Persons encountering health services in other circumstances, (28) Mental and behavioral disorders due to psychoactive substance use, (29) Disease pattern/syndrome of Oriental Medicine, (30) Hernia, (31) Persons with potential health hazards related to communicable diseases, (32) Systemic connective tissue disorder.
5-year	(1) Age, (2) Organic, including symptomatic mental disorders, (3) Educational level, (4) Sex, (5) Acute upper respiratory infections, (6) Dorsopathies, (7) Metabolic disorders, (8) Disorders of thyroid gland, (9) Disorders of conjunctiva, (10) Disorders of lens, (11) Region of residence, (12) Mood (affective) disorders, (13) Extrapyramidal and movement disorders, (14) Diabetes mellitus, (15) Cerebral palsy and other paralytic syndromes, (16) Other degenerative diseases of the nervous system, (17) Noninflammatory disorders of female genital tract, (18) Abnormal findings on examination of blood, without diagnosis, (19) Persons encountering health services for examination and investigation, (20) Persons encountering health services in other circumstances, (21) Injuries to unspecified parts of trunk, limb, or body region, (22) Schizophrenia, schizotypal, and delusional disorders, (23) Visual disturbances and blindness, (24) Mental and behavioral disorders due to psychoactive substance use, (25) Pediculosis, acariasis, and other infestations.

**Table 8:** Dementia risk factors showing differences between the 1- and 5-year incubation period models.

Incubation Period	Dementia Risk Factors and Ranking
1-year	(1) Hypertensive diseases, (2) Diseases of esophagus, stomach, and duodenum, (3) Soft tissue disorders, (4) Other acute lower respiratory infections, (5) Cerebrovascular diseases, (6) Dermatitis and eczema, (7) Health insurance type, (8) Mycoses, (9) Other diseases of the urinary system, (10) Osteopathies and chondropathies, (11) Diseases of liver, (12) Diseases of inner ear, (13) Abnormal findings on diagnostic imaging and in function studies, without diagnosis, (14) Polyneuropathies and other disorders of the peripheral nervous system, (15) Injuries to the head, (16) Injuries to the hip and thigh, (17) Noninfective enteritis and colitis, (18) Aplastic and other anemias, (19) Disease Name of Oriental Medicine, (20) Other bacterial diseases, (21) Sequelae of infectious and parasitic diseases, (22) Abnormal findings on examination of urine, without diagnosis, (23) Demyelinating diseases of the central nervous system, (24) Poisoning by drugs, medicaments, and biological substances, (25) Inflammatory diseases of the central nervous system, (26) Disease of appendix, (27) Other congenital malformations of the digestive system.
5-year	(1) Dorsopathies, (2) Disorders of thyroid gland, (3) Region of residence, (4) Extrapyramidal and movement disorders, (5) Diabetes mellitus, (6) Other degenerative diseases of the nervous system, (7) Noninflammatory disorders of female genital tract, (8) Abnormal findings on examination of blood, without diagnosis, (9) Persons encountering health services for examination and investigation, (10) Persons encountering health services in other circumstances, (11) Injuries to unspecified parts of trunk, limb, or body region, (12) Schizophrenia, schizotypal, and delusional disorders, (13) Visual disturbances and blindness.

**Table 9:** Newly identified dementia risk factors by the incubation period model.

Incubation Period	Dementia Risk Factors and Ranking
1-year	(6) Soft tissue disorders, (13) Dermatitis and eczema, (16) Health insurance type, (19) Osteopathies and chondropathies, (23) Abnormal findings on diagnostic imaging and in function studies, without diagnosis, (26) Injuries to the hip and thigh, (29) Disease Name of Oriental Medicine, (31) Other bacterial diseases, (32) Pediculosis, acariasis, and other infestations, (33) Sequelae of infectious and parasitic diseases, (38) Disease of appendix.
3-year	(11) Osteopathies and chondropathies, (14) Injuries to the thorax, (23) Disability registration information, (24) Disorders of skin appendages, (26) Symptoms and signs involving the nervous and musculoskeletal systems, (27) Persons encountering health services in other circumstances, (29) Disease pattern/syndrome of Oriental Medicine, (31) Persons with potential health hazards related to communicable diseases, (32) Systemic connective tissue disorder.
5-year	(11) Region of residence, (17) Noninflammatory disorders of female genital tract, (18) Abnormal findings on examination of blood, without diagnosis, (19) Persons encountering health services for examination and investigation, (20) Persons encountering health services in other circumstances, (21) Injuries to unspecified parts of trunk, limb, or body region, (25) Pediculosis, acariasis, and other infestations.

**Table 10:** Common dementia risk factors shared between the 1- and 5-year incubation period model.

Incubation Period	Dementia Risk Factors
1- and 5-year	Age, Sex, Educational level, Organic, including symptomatic mental disorders, Cerebral palsy and other paralytic syndromes, Mood (affective) disorders, Metabolic disorders, Disorders of lens, Disorders of conjunctiva, Mental and behavioral disorders due to psychoactive substance use, Pediculosis, acariasis, and other infestations, Acute upper respiratory infections.

**Table 11:** Changes in the distribution of chronic diseases among common dementia risk factors.

**Stage**	**Organic, Including Symptomatic Mental Disorders**		**Cerebral Palsy and Other Paralytic Syndromes**		**Mood (Affective) Disorders**	
	**IP 5-Year (%)**	**IP 1-Year (%)**	**IP 5-Year (%)**	**IP 1-Year (%)**	**IP 5-Year (%)**	**IP 1-Year (%)**
Preclinical	81.0	9.2	96.5	94.9	87.2	81.9
Minor	9.8	52.8	1.9	3.1	8.2	11.4
Severe	4.7	16.4	1.3	1.5	3.3	3.7
Critical	4.5	21.5	0.3	0.5	1.3	3.0
**Stage**	**Metabolic Disorders**		**Disorders of Lens**		**Disorders of Conjunctiva**	
	**IP 5-Year (%)**	**IP 1-Year (%)**	**IP 5-Year (%)**	**IP 1-Year (%)**	**IP 5-Year (%)**	**IP 1-Year (%)**
Preclinical	85.4	84.2	72.9	87.3	72.3	84.7
Minor	6.7	9.1	22.8	11.5	24.9	14.2
Severe	4.5	4.3	4.0	1.0	2.7	1.1
Critical	3.3	2.4	0.3	0.2	0.1	0.0

Abbreviations: IP: Incubation Period.

**Table 12:** Changes in the distribution of acute diseases among common dementia risk factors.

Stage	Mental and Behavioral Disorders Due to Psychoactive Substance Use		Pediculosis, Acariasis, and Other Infestations		Acute Upper Respiratory Infections	
	IP 5-Year (%)	IP 1-Year (%)	IP 5-Year (%)	IP 1-Year (%)	IP 5-Year (%)	IP 1-Year (%)
Preclinical	99.3	98.3	99.0	97.8	40.9	67.1
Minor	0.3	1.0	0.3	1.0	47.3	27.9
Severe	0.2	0.5	0.5	1.0	11.0	4.9
Critical	0.2	0.2	0.2	0.2	0.9	0.2

Abbreviations: IP: Incubation Period.

## Data Availability

The data presented in this study are available on request from the corresponding author.
